# Activated Platelets Convert CD14^+^CD16^-^ Into CD14^+^CD16^+^ Monocytes With Enhanced FcγR-Mediated Phagocytosis and Skewed M2 Polarization

**DOI:** 10.3389/fimmu.2020.611133

**Published:** 2021-01-07

**Authors:** Su Jeong Lee, Bo Ruem Yoon, Hee Young Kim, Su-Jin Yoo, Seong Wook Kang, Won-Woo Lee

**Affiliations:** ^1^ Laboratory of Autoimmunity and Inflammation (LAI), Department of Biomedical Sciences, Seoul National University College of Medicine, Seoul, South Korea; ^2^ BK21Plus Biomedical Science Project, Seoul National University College of Medicine, Seoul, South Korea; ^3^ Department of Microbiology and Immunology, Seoul National University College of Medicine, Seoul, South Korea; ^4^ Institute of Infectious Diseases, Seoul National University College of Medicine, Seoul, South Korea; ^5^ Department of Internal Medicine, Chungnam National University School of Medicine, Daejeon, South Korea; ^6^ Cancer Research Institute, Seoul National University College of Medicine, Seoul National University Hospital Biomedical Research Institute, Seoul, South Korea; ^7^ Ischemic/Hypoxic Disease Institute, Seoul National University College of Medicine, Seoul, South Korea; ^8^ Seoul National University Hospital Biomedical Research Institute, Seoul, South Korea

**Keywords:** TGF-β, IL-6, phagocytosis, platelet, CD14^+^CD16^+^ monocytes, M2 macrophages, rheumatoid arthritis, MerTK

## Abstract

Monocytes are important cellular effectors of innate immune defense. Human monocytes are heterogeneous and can be classified into three distinct subsets based on CD14 and CD16 expression. The expansion of intermediate CD14^+^CD16^+^ monocytes has been reported in chronic inflammatory diseases including rheumatoid arthritis (RA). However, the mechanism underlying induction of CD16 and its role in monocytes remains poorly understood. Here, we demonstrate that activated platelets are important for induction of CD16 on classical CD14^+^CD16^-^ monocytes by soluble factors such as cytokines. Cytokine neutralization and signaling inhibition assays reveal that sequential involvement of platelet*-*derived TGF-β and monocyte-derived IL-6 contribute to CD16 induction on CD14^+^CD16^-^ monocytes. Activated platelet-induced CD16 on monocytes participates in antibody-dependent cellular phagocytosis (ADCP) and its level is positively correlated with phagocytic activity. CD14^+^CD16^-^ monocytes treated with activated platelets preferentially differentiate into M2 macrophages, likely the M2c subset expressing CD163 and MerTK. Lastly, the amount of sCD62P, a marker of activated platelets, is significantly elevated in plasma of RA patients and positively correlates with clinical parameters of RA. Our findings suggest an important role of activated platelets in modulating phenotypical and functional features of human monocytes. This knowledge increases understanding of the immunological role of CD14^+^CD16^+^ cells in chronic inflammatory diseases.

## Introduction

Monocytes are circulating blood leukocytes typically regarded as systemic precursors of macrophages and dendritic cells (DCs) (1–3). Besides their primary role as precursors, monocytes also act as important innate effectors in the pathogenesis of various inflammatory diseases as well as in the inflammatory response against infectious pathogens through phagocytosis, production of reactive oxygen species (ROS), secretion of proinflammatory soluble factors, and the activation of adaptive immunity (4, 5). In humans, peripheral monocytes are heterogeneous and classified into three functionally distinct subsets depending on the expression of CD14, a coreceptor for LPS, and CD16 (also known as FcγRIII). These include classical CD14^+^CD16^-^, intermediate CD14^+^CD16^+^, and nonclassical CD14^dim^CD16^+^ monocytes (6–9). *In vivo* deuterium labeling experiments revealed that CD14^+^CD16^-^ monocytes have the potential to become CD14^+^CD16^+^ monocytes before finally differentiating into CD14^dim^CD16^+^ monocytes under steady state and experimental endotoxemic conditions (10). Moreover, CD16^+^ monocytes are expanded in patients with inflammatory disorders including several autoimmune diseases (11–13), and platelets are a major factor contributing to the induction of CD16 expression on human monocytes (14). However, the mechanisms underlying the induction of CD16 are not fully understood.

Platelets are circulating, tiny, anucleate cells that play a prominent role in hemostasis and thrombosis (15). However, platelets are also involved in aiding and modulating inflammatory reactions and immune responses. This occurs through immune ligands and receptors on the platelet surface and through release of an abundance of secretory molecules, including inflammatory mediators and cytokines (16). Upon activation, platelets change their shape and form aggregates. In addition, P-selectin (CD62P) expressed on activated platelets mediates the formation of monocyte-platelet aggregates (MPAs), which is an essential pathophysiological mechanism that mediates the induction of inflammatory events by activated platelets (17, 18). Several studies have shown increased levels of circulating MPAs in the peripheral blood of patients with atherosclerosis, type I diabetes, and end-stage renal diseases (19–22). In several autoimmune diseases, including rheumatoid arthritis (RA) and systemic lupus erythematosus (SLE), platelets are considered active players that produce serotonin and IL-1-containing microparticles (23–26). Further, platelets promote macrophage polarization toward the proinflammatory phenotype in response to LPS stimulation resulting in increased survival of septic mice (27), whereas platelet-lymphocyte interactions mediate anti-inflammatory events in rheumatoid arthritis (RA) (28). Together this suggests platelets play a regulatory role in innate as well as adaptive immune responses (15, 29).

In our previous study we demonstrated that CD14^+^CD16^+^ monocytes are markedly expanded in peripheral blood and synovial fluid of RA patients. Further, CD16 expression on CD14^+^ monocytes is induced by TGF-β without additive effects of co-treatment with IL-1β, TNF-α or IL-6, which are typical proinflammatory cytokines produced by activated monocytes (11). Given the involvement of platelets in the pathophysiology of RA and their role as a major reservoir of TGF-β (30), we sought to investigate the underlying mechanisms of CD16 induction on monocytes and the immunological role of this receptor under co-culture conditions with activated, autologous platelets.

In the present study, we demonstrate that CD16 expression is induced by exposure to the cytokine milieu generated in monocyte and ADP-activated platelet co-cultures. Exogenous cytokine treatment and neutralization assay showed that both platelet-derived TGF-β and monocyte-derived IL-6 are sequentially involved in the induction of CD16 expression on purified CD14^+^CD16^-^ monocytes. Induced CD16 participates in IgG-mediated phagocytosis, as shown by the correlation between the level of CD16 expression by monocytes co-cultured with activated platelets and the CD16-dependent uptake of latex beads coated with FITC-labeled IgG. In addition, monocytes pretreated with activated platelets preferentially differentiate into M2c-like macrophages in the presence of M-CSF. Lastly, the amount of sCD62P, a marker of platelet activation, was found to be significantly elevated in plasma of RA patients compared with that of healthy controls and positively correlated with clinical parameters of RA patients. These findings underscore the important role of activated platelets in modulating phenotypical and functional features of human monocytes. Together these findings increase understanding of the immunological role of CD14^+^CD16^+^ monocytes in various inflammatory disorders.

## Materials and Methods

### Cell Preparation

The study protocols were approved by the institutional review board (IRB) of Seoul National University Hospital and Chungnam National University Hospital. Peripheral blood of RA patients and healthy controls (HCs) was drawn after obtaining written, informed consent. The methods were performed in accordance with the approved guidelines. The patient characteristics of RA patients enrolled in this study are summarized in **Table 1**. To obtain platelets, platelet-rich plasma (PRP) was prepared from whole blood by centrifugation at 190 *g* for 15 min at room temperature (RT). Subsequently, platelet pellet was prepared from PRP by centrifugation at 2,400 *g* for 5 min and was resuspended with 25mM HEPES-buffered Tyrode’s solution (Sigma-Aldrich, St. Louis, MO). Peripheral blood mononuclear cells (PBMC) were isolated from blood by density gradient centrifugation (Bicoll separating solution; BIOCHROM Inc., Cambridge, UK). To purify CD14^+^CD16^-^ monocytes, CD16^+^ monocytes were negatively depleted from PBMC with CD16^+^ Monocyte Isolation Kit (Miltenyi Biotec Inc., Auburn, CA) and CD14^+^ monocytes were positively purified from CD16^+^ cell-depleted PBMC using anti-CD14 microbeads (Miltenyi Biotec Inc.).

**Table 1:** The characteristics of RA patients.

Patient Characteristics (Value)	Age (years), means ± SD	57.84 ± 8.81
	Sex (female/male), *N* (%)	24/8 (75.00/25.00)
	Disease duration, months	5.48 ± 5.12
	Rheumatoid factor – no. of positive (%)	28/32 (87.50)
	Rheumatoid factor titer (IU/ml), means ± SD	122.90 ± 87.70
	Anti-citrullinated protein antibody, no. of positive (%)	25/32 (78.13)
	Anti-citrullinated protein antibody titer (IU/ml), means ± SD	357.88 ± 161.59
	ESR (mm/h), means ± SD	26.81 ± 23.98
	CRP (mg/dl), means ± SD	2.00 ± 6.17
	DAS28 ESR, means ± SD	3.36 ± 1.62
	DAS28 CRP, means ± SD	2.42 ± 1.29
Medications [No. (%)]	Steroid (Prednisolone)	25/32 (78.13)
	Methotrexate	27/32 (84.38)
	Sulfasalazine	10/32 (31.25)
	Hydroxychloroquine	15/32 (46.88)
	Leflunomide	1/32 (3.13)
	TNF inhibitor (Infliximab)	1/32 (3.13)

ESR, erythrocyte sedimentation rate.

CRP, C-reactive protein.

DAS28, Disease Activity Score-28.

DAS28-ESR, DAS28 for RA based on ESR.

DAS28-CRP, DAS28 for RA based on CRP.

### Co-Culture of Monocytes and Activated Platelets

Purified CD14^+^CD16^-^ monocytes were cultured in RPMI 1640 medium supplemented with 10% charcoal stripped fetal bovine serum, 1% penicillin/streptomycin, and 1% l-glutamine. Platelets were activated with ADP (10 μg/ml) for 5 min at RT. CD14^+^CD16^-^ monocytes were co-cultured with activated platelets at 1:100 ratio for 18 h at 37°C in polystyrene tubes. In some experiments, CD14^+^CD16^-^ monocytes and activated platelets were placed in lower- and upper-chamber of Transwell cell culture plate (0.4 μm pore size) (Corning-Costar, Lowell, MA), respectively and cultured for 18 h at 37°C. To prepare human monocyte-derived macrophage (HMDM), purified monocytes were pretreated for 18 h with or without ADP-activated platelets and were differentiated into macrophages for 6 days in the presence of recombinant human M-CSF (50 ng/ml; PeproTech, Rocky Hill, NJ) without washing platelets.

### Cytokine Neutralization Assay and Signaling Inhibition Assay

Purified CD14^+^CD16^-^ monocytes were pre-incubate for 30 min at 37°C with anti-IL-6, anti-TGF-β 1,2,3, or anti-IL-10 neutralizing Ab (all from R&D systems, Minneapolis, MN) to examine the effect of above cytokines on CD16 induction by activated platelets. In some experiments, CD14^+^CD16^-^ monocytes were pre-incubate for 30 min at 37°C with 5,15-DPP (STAT3 inhibitor VIII), SIS3 (SMAD3 inhibitor), or SB431542 (TGF-βRI inhibitor) (all from MERCK, Burlington, MA) to examine the effect of TGF-β and IL-6-mediated signal transduction on CD16 induction by activated platelets.

### Flow Cytometric Analysis

Cultured monocytes and HMDM were stained for 30 min at 4°C with following fluorochrome-conjugated Abs: PE-Cy5-anti-HLA-DR, FITC- or APC-Cy7-anti-CD14, PE-anti-CD16, APC-anti-CD62P (all from BD Bioscience, San Jose, CA), and APC-anti-CD80 (BioLegend, San Jose, CA). The stained cells were acquired using a BD LSR Fortessa (BD Bioscience) and analyzed using Flowjo software (Tree Star, Ashland, OR).

### Quantitative RT-PCR

Total RNA was extracted from freshly isolated or cultured cells using TRIzol reagents (Life technologies, Grand Island, NY), and cDNA was synthesized by GoScript reverse transcription system (Promega, Madison, WI). Real-time quantitative RT-PCR was performed in triplicates on a 7500 PCR system (Applied Biosystems, Grand Island, NY) using following primers: *CD16*: 5′-GCTCCGGATATCTTTGGTGA-3′ and 5′- TTCCAGCTGTGACACCTCAG -3′; *TGF-β*: 5′- AAGTGGACATCAACGGGTTC -3′ and 5′-GTCCTTGCGGAAGTCAATGT-3′; *IL-6*: 5′-AGGAGACTTGCCTGGTGAAA-3′ and 5′-CAGGGGTGGTTATTGCATCT-3′; *IL-10*: 5′-TGCCTTCAGCAGAGTGAAGA-3′ and 5′-GGTCTTGGTTCTCAGCTTGG-3′; *CD80*: 5′-GGGAAAGTGTACGCCCTGTA-3′ and 5′-GCTACTTCTGTGCCCACCAT-3′; *CXCL10*: 5′-CAGCAGAGGAACCTCCAGTC-3′ and 5′-CAAAATTGGCTTGCAGGAAT-3′; *TNF-α*: 5′-AGCCCATGTTGTAGCAAACC-3′ and 5′-TGAGGTACAGGCCCTCTGAT-3′; *MRC1*: 5′-TGACACACTTTTGGGGATCA-3′ and 5′-AAACTTGAACGGGAATGCAC-3′; *Dectin-1*: 5′-GGGCTCTCAAGAACAATGGA-3′ and 5′-CCAAGCATAGGATTCCCAAA-3′; CD163: 5′-TTTGCTCAAAGGGAGCAGAT-3′ and 5′-GTTGGACATCCCAGTTGCTT-3′; *MerTK*: 5′-GGGTGAAGGAGAGTTTGGGTC-3′ and 5′-ACGCTGCCTCACTGAGAAAC-3′; and β*-actin*: 5′-GGACTTCGAGCAAGAGATGG-3′ and 5′-AGCACTGTGTTGGCGTACAG-3′. The levels of gene expression were normalized to the expression of β-actin. The comparative CT method (*ΔΔ*CT) was used for the quantification of gene expression.

### Enzyme-Linked Immunosorbent Assay

The amount of TGF-β, IL-6, and IL-10 in culture supernatant and soluble CD62P in plasma of RA patients and HCs was quantified by enzyme-linked immunosorbent assay (ELISA) kits (all from Thermo Fisher Scientific, Waltham, MA). The measurement of OD (Optical density) was performed using the infinite 200 pro multimode microplate reader (Tecan Group Ltd., Seestrasse, Switzerland).

### Immunoblot Analysis

Total proteins were prepared by RIPA buffer (150 mM NaCl, 10 mM Na_2_HPO_4_, pH 7.2, 1% Nonidet P-40, and 0.5% deoxycholate) containing PMSF (phenylmethylsulfonyl fluoride) (Millipore Sigma, Burlington, MA), EDTA, and protease and phosphatase inhibitor cocktail (Thermo Fisher scientific). Cell lysates were separated on an 8–10% SDS-PAGE gel and blotted onto a PVDF membrane (Bio-Rad, Hercules, CA). The membrane was incubated overnight at 4°C with rabbit anti-human pSTAT3, anti-STAT3, anti-pSMAD3 or anti-SMAD3 polyclonal Ab (all from Cell Signaling Technology, Danvers, MA), followed by incubation with the HRP-conjugated secondary Abs for 1 hr. The membranes were developed by SuperSignal West Femto Maximum Sensitivity substrate kit (Thermo Fisher Scientific, Waltham, MA).

### Phagocytosis Assay

CD16-dependent phagocytic activity of monocytes co-cultured with platelets were assessed by flow cytometry using Phagocytosis Assay Kit (Cayman Chemical Company, Ann Arbor, MI). Cultured monocytes were pre-incubated at 37°C for 30 min with anti-CD64 and anti-CD32 neutralizing Abs (all from BioLegend) in the presence or absence of anti-CD16 neutralizing Ab (BD Bioscience). After washing, Latex beads coated with FITC labeled rabbit IgG were added at 1:400 ratio into the cultured monocytes, followed by incubation at 37°C for 15 min. The phagocytic activity was evaluated using a BD LSR Fortessa (BD Bioscience).

### Statistics

A paired *t*-test, unpaired *t*-test, or Pearson correlation analysis was done to analyze data using Prism 7 software (GraphPad Software Inc., La Jolla, CA) as indicated in the figure legends. *P*-values of less than 0.05 were considered statistically significant.

## Results

### Induction of CD16 on CD14^+^CD16^-^ Monocytes by Activated Platelets

**Figure 1:**
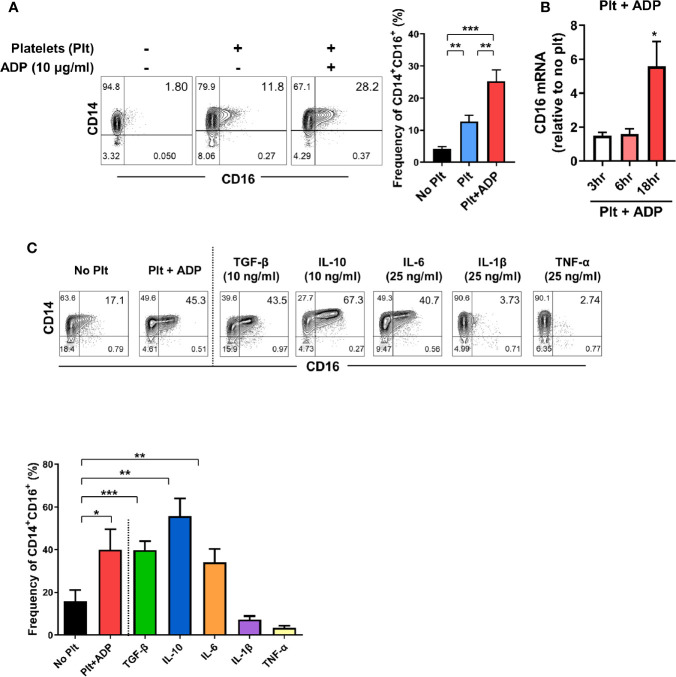
Activated platelets induce CD16 expression on CD14^+^CD16^-^ monocytes. **(A)** Representative contour plots of CD16 expression on monocytes co-cultured with or without activated platelets (right). Highly purified CD14^+^CD16^-^ monocytes were co-cultured for 18 h with resting platelets or ADP-activated platelets. Frequencies (%) of CD14^+^CD16^+^ monocytes were analyzed using flow cytometry (left) (*n* = 7). **(B)** Quantitative RT-PCR analysis of CD16 in monocytes co-cultured with activated platelets for the indicated times. mRNA expression of CD16 was normalized to that in monocytes incubated without platelets at the same time points (*n* = 5 or 6). **(C)** Representative contour plot of CD16 expression on monocytes co-cultured with activated platelets for 18 h or treated with the indicated cytokines for 18 h (upper panel). Frequencies (%) of CD14^+^CD16^+^ monocytes under the indicated conditions were analyzed using flow cytometry (lower panel) (*n* = 4). Bars show the mean ± S.E.M. *p < 0.05, **p < 0.01, and ***p < 0.001 by two-tailed paired *t*-test.

The expansion of CD14^+^CD16^+^ monocytes has been reported in a variety of inflammatory disorders including rheumatoid arthritis (RA) and inflammatory bowel disease (IBD) (11, 12, 31), implying an important role of these cells in disease pathogenesis. Our previous study demonstrated that TGF-β predominantly induces CD16 expression on conventional CD14^+^ monocytes isolated from healthy controls (11). Considering that platelets are a major reservoir of TGF-β, we first tested whether platelets induce CD16 expression on human monocytes. Highly purified CD14^+^CD16^-^ monocytes were co-cultured with autologous platelets pretreated with or without adenosine diphosphate (ADP) to activate platelets. Our FACS analysis using CD62P, a marker of platelet activation, showed that around 10% of platelets were spontaneously activated before adding ADP, probably by physical stress during isolation. However, ADP treatment markedly induced the activation of platelets (**Supplementary Figure 1**). We found that platelets significantly increased the expression of CD16 on CD14^+^ monocytes as previously depicted (14). This induction was intensified by ADP-treatment of platelets (**Figure 1A**) in a transcription-dependent manner (**Figure 1B**), with markedly elevated CD16 mRNA expression 18 h after co-culture. Monocytes treated with ADP alone did not induce CD16 expression on CD14^+^CD16^-^ monocytes (**Supplementary Figure 2**) although it has been known that P2Y12, a receptor for ADP, is expressed on THP-1 human monocyte cells (32). Activated platelets give rise to an increase of the CD14^+^CD16^+^ subset, but had no effect on the appearance of nonclassical CD14^dim^CD16^+^ monocytes (**Figure 1A**). Although stimulated platelets are a main source of TGF-β, the adherence of platelets to monocytes is also known to influence the production of cytokines such as IL-10 and TNF-α (33). Therefore, we extended our analysis to examine which cytokines, in addition to TGF-β, induce CD16 expression and compared these findings with the platelet-treatment group. In agreement with previous studies, CD14^+^ monocytes treated with exogenous TGF-β and IL-10 exhibited significantly enhanced CD16 expression (**Figure 1C**) (11, 14, 33, 34). However, the major pro-inflammatory cytokines produced by activated monocytes, IL-1β and TNF-α, exhibited no effect on CD16 induction. Of note, in contrast to other monocyte-derived pro-inflammatory cytokines, such as IL-1β and TNF-α, IL-6 was found to induce CD16 to a degree similar to that induced by TGF-β or platelet treatment (**Figure 1C**). These findings demonstrate that cytokines derived from monocyte-platelet co-cultures are responsible for induction of CD16 on conventional CD14^+^ human monocytes.

### TGF-β and IL-6 Are Involved in Activated Platelet-Mediated Induction of CD16 on Monocytes

**Figure 2:**
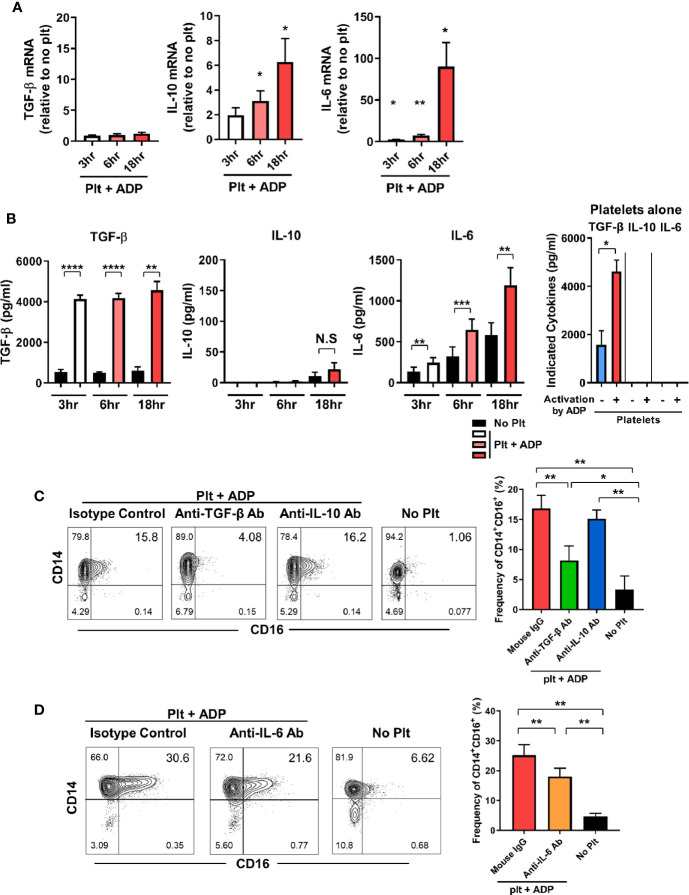
TGF-β and IL-6 are responsible for activated platelet-mediated induction of CD16 on monocytes. **(A)** Quantitative RT-PCR analysis of TGF-β, IL-10, and IL-6 in monocytes co-cultured with activated platelets at the indicated time points. mRNA expression of each gene was normalized to that in monocytes incubated without platelets at the same time points (*n* = 5 or 6). **(B)** The amount of TGF-β, IL-10, and IL-6 in supernatants of monocytes co-cultured with or without ADP-activated platelets (*n* = 6 or 7) or unstimulated platelets (*n* = 3) were quantified (ELISA). **(C, D)** Representative contour plot of CD16 expression on monocytes co-cultured with activated platelets in the presence of anti-TGF-β (10 μg/ml), anti-IL-10 (10 μg/ml), or anti-IL-6 (10 μg/ml) neutralizing antibodies (left panels of **C** and **D**). Purified CD14^+^CD16^-^ monocytes were incubated with each neutralizing antibody for 30 min, followed by addition of ADP-activated platelets into the culture. Frequencies (%) of CD14^+^CD16^+^ monocytes under the indicated conditions were analyzed using flow cytometry (right panels of **C** and **D**) (*n* = 4 or 5). Bars show the mean ± S.E.M. *p < 0.05, **p < 0.01, ***p < 0.005, and ****p < 0.001 by two-tailed paired *t*-test. NS, not significant.

We next investigate whether the induction of CD16 expression on monocytes by activated platelets is directly attributable to endogenously secreted TGF-β, IL-10, or IL-6 in the co-culture. The expression of IL-10 and IL-6 mRNA was significantly increased in a time-dependent manner during the co-culture, whereas the TGF-β mRNA level was not changed until 18 h after co-culture (**Figure 2A**). ELISA revealed that TGF-β was mainly secreted by activated platelets and was minimally produced by monocytes cultured with activated platelets and thus, a high level of TGF-β was maintained over time (**Figure 2B**). This finding was confirmed using a transwell system in which direct contact between monocytes and activated platelets mediated by CD62P was found to be dispensable for CD16 induction at 18 h after co-culture (**Supplementary Figure 3**). In stark contrast to TGF-β, platelets have no ability to produce IL-10 or IL-6 regardless of their activation state. Consistent with its mRNA expression, the amount of IL-6 in the co-culture supernatant increased greatly over time compared with the culture of monocytes alone (**Figure 2B**). In contrast, IL-10 was detected in the supernatant a later time-point, although at a relatively low level, and the amount of IL-1β released from the co-culture was minimal (< 10 pg/ml). Moreover, the production of IL-1β and TNF-α was comparable between co-culture of monocytes and platelets and culture of monocytes alone (data not shown). This suggests that TGF-β and IL-6, not IL-10, are the main contributors to the induction of CD16 expression on CD14^+^ monocytes co-cultured with activated platelets. To confirm this finding, purified CD14^+^ monocytes were cultured with ADP-activated platelets in the presence of neutralizing antibodies for cytokines related to induction of CD16. As expected, neutralization of TGF-β and IL-6 significantly diminished the induction of CD16 expression, whereas no inhibitory effect on CD16 expression by IL-10 was observed (**Figures 2C, D**). Our data suggest that endogenously secreted TGF-β and IL-6 are predominantly involved in activated platelet-mediated induction of CD16 on monocytes.

### Activated Platelet-Induced CD16 Expression Is Mediated by SMAD3 and STAT3

**Figure 3:**
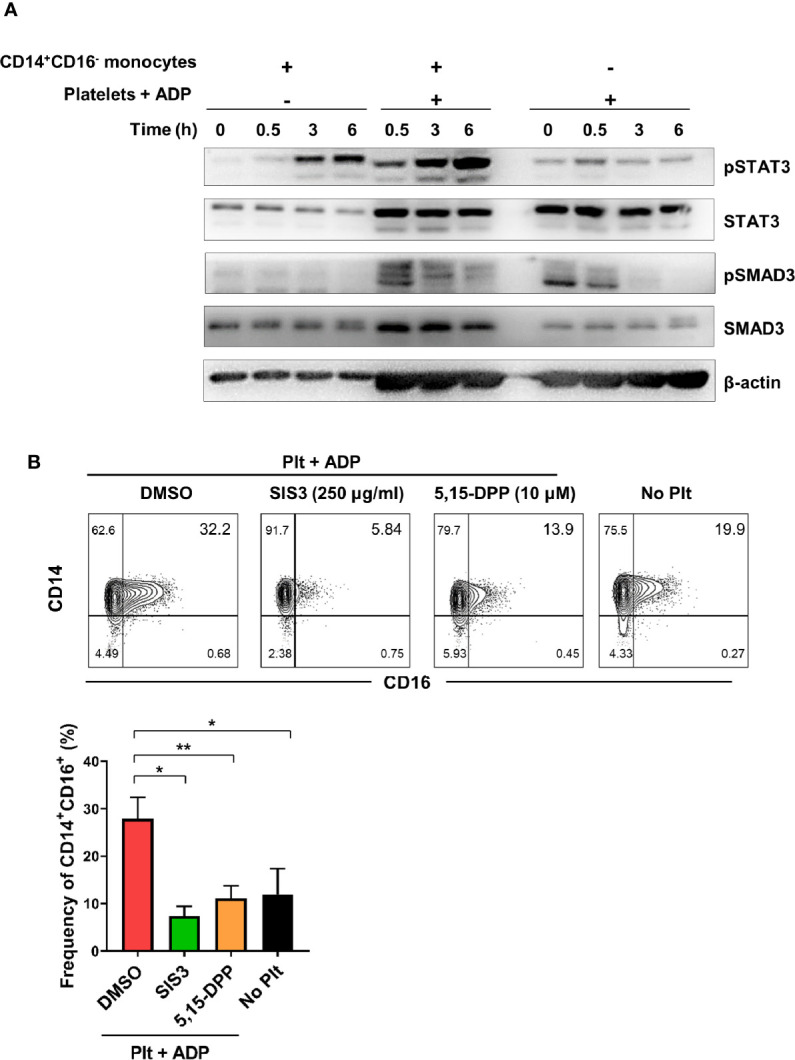
Activated platelet-induced CD16 expression is mediated by phospho-SMAD3 and phospho-STAT3. **(A)** Immunoblot analysis of phosphorylated and total protein expression of STAT3 and SMAD3 in cell lysates from activated platelets alone, monocytes co-cultured with ADP-activated platelets, and monocytes alone at the indicated time points (*n* = 3). **(B)** Representative contour plot of CD16 expression on monocytes co-cultured for 18 h with activated platelets in the presence of SIS3 (SMAD3 inhibitor) or 5,15-DPP (STAT3 inhibitor) (upper panel). Frequencies (%) of CD14^+^CD16^+^ monocytes treated with SIS3 or 5,15-DPP were analyzed using flow cytometry (lower panel) (*n* = 4). 0.1% DMSO was used as a vehicle. Bars show the mean ± S.E.M. *p < 0.05 and **p < 0.01 by two-tailed paired *t*-test.

To further investigate the molecular mechanisms underlying activated platelet-mediated induction of CD16 by monocytes, we sought to investigate whether SMAD3 and STAT3, which are directly phosphorylated by receptor binding of TGF β and IL-6, respectively, were involved in CD16 induction. Due to the technical difficulty of separating monocytes and platelets during the co-culture, the phosphorylation levels of STAT3 and SMAD3 were analyzed in the co-culture samples and were compared with monocytes or platelet-alone culture groups. STAT3 phosphorylation increased in the co-culture supernatant starting at 3 h, at which time IL-6 was also increased (**Figure 2B**) (**Figure 3A**). SMAD3 was immediately phosphorylated in platelets upon stimulation with ADP, likely in an autocrine manner. When co-cultured with monocytes, activated platelets enhanced the level of SMAD3 phosphorylation over time (**Figure 3A**). These findings were corroborated by a signal transduction inhibitor assay. SIS3, a SMAD3 inhibitor, and 5,15-DPP, a STAT3 inhibitor, significantly suppressed activated platelet-mediated induction of CD16 by monocytes (**Figure 3B**). Our data illustrate that activated platelets induce TGF-β and IL-6 *via* the SMAD3 and STAT3 pathways, respectively, which are responsible for the induction of CD16 on monocytes.

### Activated Platelet-Induced CD16 Expression Is Involved in CD16-Dependent Phagocytosis in Monocytes

**Figure 4:**
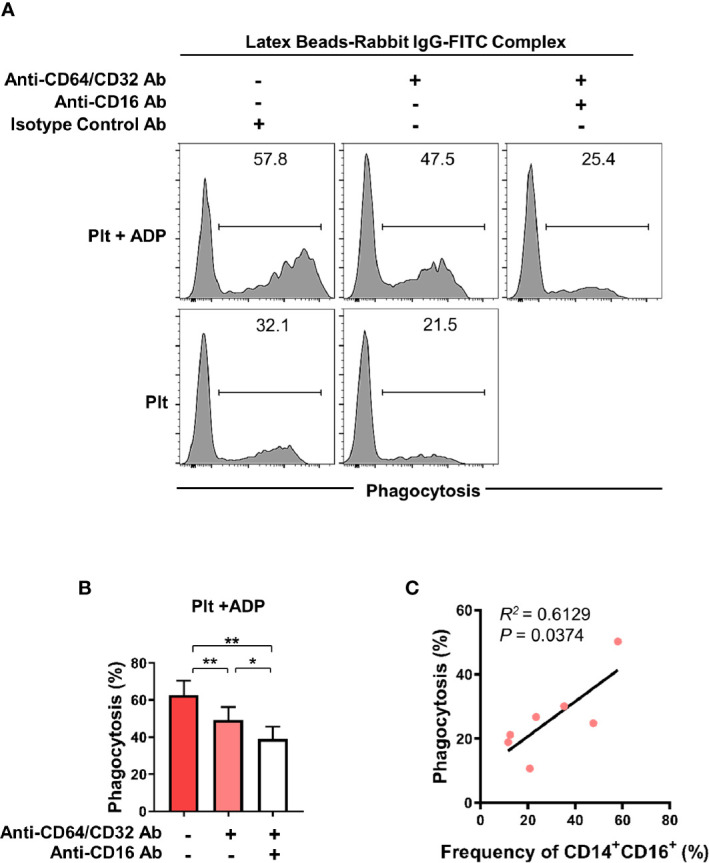
Activated platelet-induced CD16 expression on monocytes is involved in CD16-dependent phagocytosis. Purified CD14^+^CD16^-^ monocytes were co-cultured with resting platelets or APD-activated platelets for 18 h Monocytes were incubated for 15 min with latex beads coated with FITC-labeled rabbit IgG. The phagocytic activity of monocytes was analyzed using flow cytometry. **(A)** Representative histogram plots of latex bead-based phagocytic activity of monocytes. To evaluate CD16-dependent phagocytic activity, CD64 and CD32, both Fcγ receptors, were blocked for 30 min by anti-CD64 and anti-CD32 neutralizing antibodies (10 μg/ml of each antibody) and their phagocytic activity was quantified in monocytes co-cultured with resting platelets or ADP-activated platelets and with or without anti-CD16 neutralizing antibody (10 μg/ml). **(B)** Frequency (%) of monocyte phagocytosis of latex beads coated with FITC-labeled rabbit IgG under the indicated conditions. (*n* = 6) **(C)** Correlation between CD16-dependent phagocytic activity and the frequency of CD14^+^CD16^+^ monocytes after 18 h-co-culture with ADP-activated platelets (*n* = 7). *P* value was obtained using the Pearson correlation analysis. Bars show the mean ± S.E.M. *p < 0.05 and **p < 0.01 by two-tailed paired *t*-test.

CD16 is an Fc gamma receptor (FcγR) that binds IgG molecules through their Fc portion. FcγRs recognize IgG-coated targets, such as opsonized pathogens or immune complexes (ICs), and provoke antibody-mediated effector functions in innate immune cells. These include antibody-dependent cell-mediated cytotoxicity (ADCC), antibody-dependent cellular phagocytosis (ADCP), and complement-dependent cytotoxicity (CDC) (35, 36). To assess the phagocytic activity of CD16-expresssing monocytes, we utilized a flow cytometry-based phagocytosis assay with latex beads coated with FITC-labeled rabbit IgG. To rule out CD64 and CD32-mediated phagocytosis, monocytes were pretreated with anti-CD64/32 neutralizing antibodies and their phagocytic activity was quantified following culture with platelets or ADP-treated platelets (**Figure 4A** and **Supplementary Figure 4**) with or without anti-CD16 neutralizing antibody (**Figures 4A, B**). Monocytes co-cultured with ADP-activated platelets showed significantly higher phagocytic activity than those incubated with resting platelets. Furthermore, blockade of CD16 with a neutralizing Ab revealed that CD16 partially contributes to the enhanced phagocytic activity of monocytes co-cultured with activated platelets. As seen in **Figure 4C**, the frequency of the CD14^+^CD16^+^ subset 18 h after co-culture significantly correlated with phagocytic activity (**Figure 4C**, *p* < 0.05). These data demonstrate that activated platelet-mediated induction of CD16 contributes to CD16-dependent phagocytosis by monocytes.

### CD14^+^ Monocytes Treated With Activated Platelets Preferentially Differentiate Into M2 Macrophages

**Figure 5:**
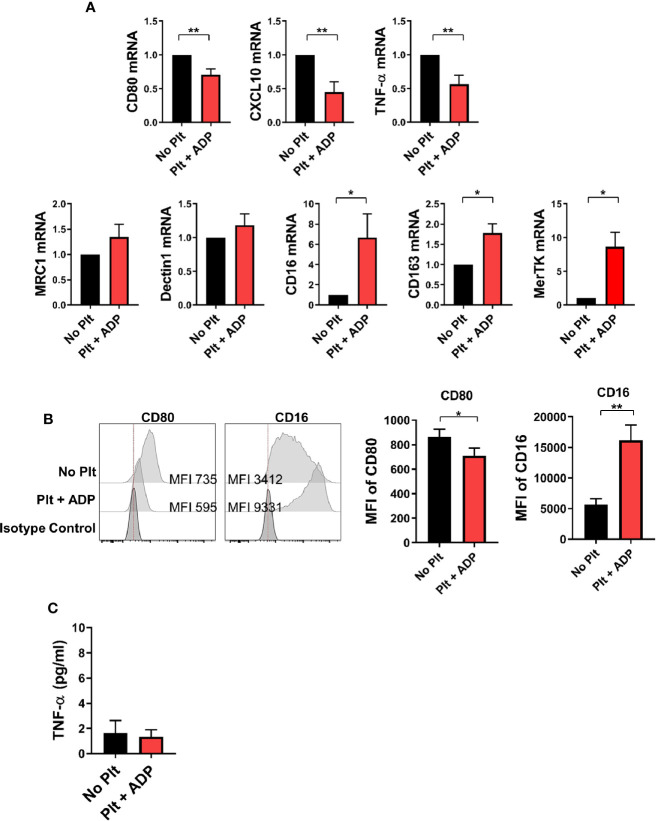
CD14^+^ monocytes treated with activated platelets preferentially differentiate into M2 macrophages. Purified CD14^+^ CD16^-^ monocytes were pre-co-cultured with ADP-activated platelets for 18 h followed by stimulation with M-CSF (50 ng/ml) for 6 days to induce differentiation into macrophages. **(A)** Quantitative RT-PCR analysis of typical M1- (upper panel) and M2-related genes (lower panel) in human monocyte-derived macrophages (HMDMs) (*n* = 5 ~ 8). **(B)** Representative histogram plots of CD80 and CD16 expression in HMDMs differentiated under the indicated conditions (left panel). Mean fluorescent intensities (MFIs) of CD80 and CD16 expression on HMDMs pre-cultured with ADP-activated platelets compared with their expression on HMDMs cultured without platelets (right panel) (*n* = 6). **(C)** TNF-α in culture supernatants (ELISA) (*n* = 9). Bars show the mean ± S.E.M. *p < 0.05 and **p < 0.01 by two-tailed paired *t*-test.

Depending on the microenvironmental stimuli and signals, circulating monocytes can differentiate into macrophages with functional heterogeneity (37–39). To examine whether the cytokine milieu generated by monocyte-platelet co-culture influences macrophage differentiation, highly purified CD14^+^ CD16^-^ monocytes were pre-treated with ADP-stimulated platelets for 18 h followed by stimulation with M-CSF for 6 days to allow differentiation into macrophages. On day 6, the expression of typical M1 and M2-related genes was analyzed by quantitative PCR in human monocyte-derived macrophages (HMDMs) in the presence of ADP-activated platelets (**Figure 5A**). The expression of M1-related genes, such as CXCL10, CD80 and TNF-α, was significantly lower in HMDMs pretreated with activated platelets than in untreated HMDMs. However, the expression of some M2-related genes, such as CD16 and CD163, was increased in HMDMs pretreated with activated platelets. Of note, MerTK, a marker of M2c macrophages, was significantly upregulated in HMDMs pretreated with activated platelets. This finding was confirmed through flow cytometric analysis and ELISA (**Figures 5B, C**). These data suggest that the cytokine milieu generated by monocyte-activated platelet co-cultures potentiates a polarization to M2 macrophages, and probably M2c macrophages.

### Clinical Relevance of Enhanced Soluble CD62P, a Marker of Platelet Activation in RA Patients

**Figure 6:**
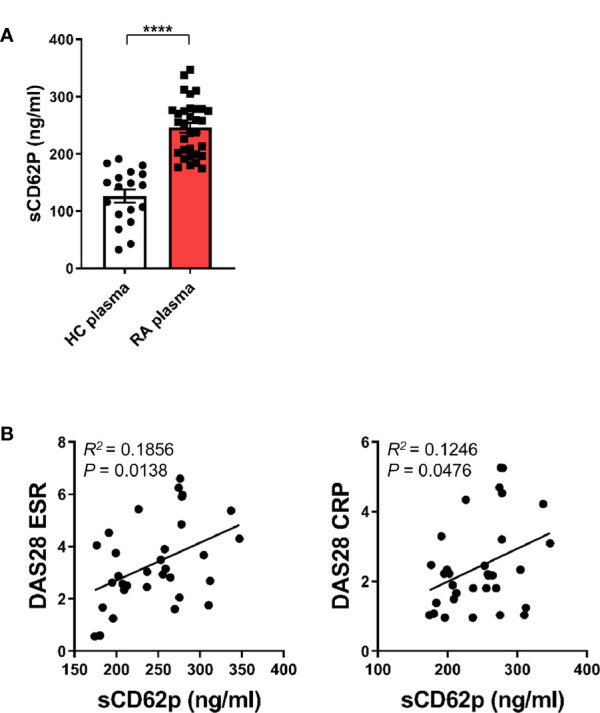
Clinical relevance of enhanced soluble CD62P, a marker of platelet activation, in RA patients. **(A)** sCD62P in plasma of HCs (*n* = 18) and RA patients (*n* = 32) was quantitated by ELISA. Scatter plots show the mean ± S.E.M. ****p < 0.0001 by two-tailed unpaired *t*-test. **(B)** Correlation between sCD62P levels in the plasma of RA patients and RA clinical parameters (*n* = 32). sCD62P levels were plotted against DAS28 ESR and DAS28 CRP. *P* value was obtained using Pearson correlation analysis.

Since our previous study revealed an expansion of CD16^+^CD14^+^ monocytes in peripheral blood of RA patients (11), we next asked whether activated platelets are associated with increased CD16 expression on peripheral monocytes in RA patients. The patient characteristics of RA patients enrolled in this study are summarized in **Table 1**. A number of soluble factors, such as platelet factor 4 (PF4), soluble CD62P (sCD62P), β-thromboglobulin, and thromboxane, are known to be released from activated platelets (40, 41). In the present study, sCD62P was quantified in plasma and compared between RA patients and age-matched healthy controls (**Figure 6A**). Consistent with previous reports (42, 43), RA patient plasma (mean ± S.D.: 245.7 ± 48.3 ng/ml, n = 32) had a significantly higher amount of sCD62P than did that of HCs (126.4 ± 48.3 ng/ml, n = 18) (*p* < 0.0001), suggesting that the presence of activated platelets is related to the expansion of CD14^+^CD16^+^ monocytes in RA patients. Lastly, we sought to examine whether elevated levels of sCD62P in plasma are associated with clinical parameters and disease activity of RA patients. The amount of sCD62P in plasma had a significant positive correlation with the Disease Activity Score-28 (DAS28) for RA based on erythrocyte sedimentation rate (DAS28-ESR) and C-reactive protein (DAS28-CRP), which represent enhanced inflammatory responses (**Figure 6B**, *p* = 0.0138 and *p* = 0.0476, respectively). Together, these findings demonstrate that increased activity of platelets, which is represented by higher level of sCD62P in plasma, is positively correlated with RA clinical parameters. This suggests a possible mechanism for the accumulation of CD14^+^CD16^+^ monocytes in RA patients.

## Discussion

Monocytes are a versatile and dynamic cell population critical for the innate immune response during infections and autoimmune diseases (2, 4, 10, 44). Surface molecule expression patterns and transcriptomic profiles demonstrate that murine equivalents of classical CD14^+^CD16^-^ and nonclassical CD14^dim^CD16^+^ subsets in humans are the proinflammatory Ly6C^hi^CX_3_CR1^mid^CCR2^+^ and patrolling Ly6C^lo^CX_3_CR1^hi^CCR2^−^ populations, respectively. The CD14^+^CD16^-^ subset mediates classical monocyte roles, such as initiation of the inflammatory response, recognition and phagocytosis of pathogens and production of proinflammatory cytokines, whereas nonclassical monocytes have anti-inflammatory properties and are involved in maintenance of vascular homeostasis (4, 44). In addition to the two monocyte subsets, a substantial number of intermediate CD14^+^CD16^+^ monocytes exist in human peripheral blood. CD14^+^CD16^+^ monocytes exhibit both phagocytic function and anti-inflammatory effects, as well as higher levels of intracellular IL-1β and TNF-α at steady state (45, 46). Research on the developmental trajectories of the three monocyte subsets in humans and in humanized mice suggests that intermediate monocytes exist in a transitory stage from classical to nonclassical monocytes at steady state and under experimental endotoxemic conditions (10). A number of studies have shown that CD14^+^CD16^+^ monocytes are expanded in peripheral blood and inflamed tissues during acute and chronic inflammation such as is seen with inflammatory bowel disease (IBD) and rheumatoid arthritis (RA) (11–13, 34). Since the murine analog to CD14^+^CD16^+^ monocytes has not been clearly identified, little is known about mechanism underlying expression of CD16 and the immunological role of intermediate monocytes.

CD16 expression has been shown to be upregulated on monocytes in response to a number of different factors including platelets and several cytokines (11, 14, 34). In agreement with an early report, our data show that activated platelets lead to a marked induction of CD16 expression on classical CD14^+^ monocytes in a transcription*-*dependent manner (**Figures 1A, B**) (14). Several studies have demonstrated that induction of CD16 expression on monocytes is mediated by activated platelet-dependent COX-2 upregulation and consequent PGE2 synthesis in monocytes. Moreover, COX-2 synthesis is modulated by adhesion and signaling in response to cytokines such as platelet-derived TGF-β1 or monocyte-derived IL-1β (47–49). Since MPAs are involved in secretion of soluble factors, including various cytokines (15, 50), we sought to determine which cytokines are capable of inducing CD16 on monocytes. Besides TGF-β1, a well-known inducer of CD16 on monocytes (11, 14), STAT3-activating cytokines IL-10 and IL-6 noticeably enhance CD16 expression, whereas IL-1β and TNF-α, major monocyte-derived pro-inflammatory cytokines, had an inhibitory effect on CD16 induction. Our previous study showed that TGF-β-induced CD16 expression on monocytes was inhibited by IL-1β and TNF-α, but not IL-6 (11). Kinetics of cytokine production by MPA reveal that TGF-β and IL-6 are involved in activated platelet-mediated induction of CD16 monocytes (**Figure 2**). Although exogenous IL-10 is a potent inducer of CD16 on monocytes, as described previously and in the present study (**Figure 1C**) (34), activated platelets have no ability to produce IL-10, nor do monocytes induce IL-10 production within 18 h after co-culture. Moreover, an IL-10 neutralization assay corroborated our findings as IL-10 blockade had no effect on CD16 induction by MPAs (**Figure 2C**). It has been demonstrated that CD14^+^CD16^+^ intermediate monocytes are induced by IL-10 and positively correlate with disease activity in rheumatoid arthritis (RA) (34). IL-10 is broadly produced by many types of immune cells (51), and it has been suggested that CD16 expression on monocytes is maintained by IL-10 produced by human naïve CD4^+^ T cells (52). Induction of CD16 by IL-10 may occur in chronic inflammatory conditions including RA or during interactions with T cells. We showed that the majority of the TGF-β was rapidly secreted from ADP-stimulated platelets in a transcription-independent manner and its production by monocytes in MPAs was minimal (**Figure 2B**). Thus, hindrance of direct contact in the transwell system had no effect on CD16 induction on monocytes. However, IL-6 was produced solely by activated monocytes in MPAs in a transcription-dependent manner as seen in the **Figure 2B**. Immunoblotting of phosphorylation of the downstream mediators, SMAD3 and STAT3, revealed an obvious difference in the pattern and kinetics of TGF-β and IL-6 production in MPA. SMAD3 in monocytes is rapidly (before 30 min) phosphorylated only when cells are co-cultured with platelets, whereas phosphorylation of STAT3 was enhanced at later time points *via* IL-6 autocrine signaling. The critical role of TGF-β and IL-6 for CD16 induction in MPA was clearly shown by inhibition with SB431542, SIS3, or 5, 15-DPP, which selectively inhibit TGF-βRI, SMAD3, and STAT3, respectively (**Supplementary Figure 5** and **Figure 3B**). It should be noted that IL-6 induced expression of COX2, which is important for CD16 induction on monocytes by activated platelets, is mediated by STAT3 in monocytic THP1 cells and prostatic tumor cells (47, 53, 54).

Recent work has highlighted a cardinal role for platelets in inflammatory and immune responses by expressing and secreting many potent immunological mediators, such as FcγRIIA (CD32), CD154, TLRs, MHC class I molecules, cytokines, chemokines, and several granules including platelet factor 4 (PF4), glutamate, serotonin, and ADP (15, 18). Activated platelets shed microparticles that bud from their membranes (30) extending the reach of the activated platelet to sites distant from the cell itself (55). Besides their well-known pathogenic role in atherosclerosis, platelets have been identified as active players in the pathogenesis of RA and SLE (30, 55, 56). Anti-citrullinated protein antibodies (ACPA), which recognize a group of post-transcriptionally modified autoantigens in RA, contribute to platelet activation and activated platelets release ADP themselves, further causing platelet activation (57). Of note, serum soluble selectin levels including sCD62P are elevated in RA and systemic sclerosis. Thus, sCD62P has been suggested as a circulating biomarker in RA with thrombocytosis, indicating the presence of a continuous underlying inflammatory stimulus (42, 43). We previously reported that CD14^+^CD16^+^, but not CD14^dim^CD16^+^, monocytes were predominantly expanded in peripheral blood and synovial fluid of RA patients compared with healthy controls (11). As seen in **Figure 6A**, the concentration of sCD62P, a biomarker of activated platelets, was significantly increased in plasma of RA patients compared with that of healthy controls, implicating activated platelets in the induction of CD16 on classical monocytes in RA. It should be noted that the expression of CD16 on CD14^+^ monocytes is induced by synovial fluid in a dose-dependent manner and this upregulation is greatly inhibited by SB431542, a TGF-βRI inhibitor (11). In RA patients, the proportion of CD14^+^CD16^+^ monocytes is positively correlated with clinical parameters, such as CRP and DAS28-ESR, and is significantly decreased after a 12-week treatment with methotrexate (MTX), a gold standard, disease-modifying anti-rheumatic drug (DMARD) (34). This is supported by our findings showing sCD62P concentration is significantly and positively correlated with RA clinical parameters including DAS28-ESR and DAS28-CRP (**Figure 6B**).

Human CD16 exists in two different isoforms, FcγRIIIA (CD16A) and FcγRIIIB (CD16B), encoded by separate genes (58). Phillips et al. showed that CD16 expressed on platelet-activated monocytes is structurally similar to the transmembrane-anchored CD16B polypeptide expressed on NK cells but is associated with the FcϵRI-γ subunit in a manner identical to that of human basophils (14). Therefore, it has been suggested that CD16 on monocytes contributes to antibody-dependent cellular cytotoxicity (ADCC) for targeting and killing virus-infected or transformed cells coated with specific antibodies (59). ADCC by CD16^+^ monocytes requires cell-cell contact facilitated *via* β2-integrins and mediated by TNF-α (59). Besides ADCC, CD16 induced by activated platelets mediates antibody-dependent cellular phagocytosis (ADCP), showing that this phagocytic activity is comparable to that of CD64/CD32-mediated phagocytosis in monocytes (**Figure 4B**). Our data illustrate that the phagocytic capacity largely depends on the platelet activity and the proportion of CD16+CD14+ monocytes induced (**Figures 4B, C**). Given that ADCC, ADCP, and complement-dependent cytotoxicity (CDC) are FcR-mediated effector functions which contribute to removal of antibody-opsonized target cells or molecules, induction of CD16 on monocytes is likely involved in regulation of immune responses and inflammatory reactions. Although not examined in our study, expanded CD14^+^CD16^+^ monocytes in RA may result in an increased responsiveness to immune complex(IC)-stimulation (60). Given that IgG-containing immune complexes (IC) including those containing rheumatoid factors (RFs) and cyclic citrullinated peptide (CCP) autoantibodies are found abundantly in serum and synovial fluid of patients with RA, it could be one potential pathophysiological role of intermediate CD14^+^CD16^+^ monocytes in RA.

A large number of circulating monocytes migrate into an inflamed tissue and generally differentiate into inflammatory macrophages during an inflammatory reactions (6, 61). Macrophages can be phenotypically and functionally polarized into proinflammatory M1 or anti-inflammatory/pro-resolving M2 macrophages depending on the surrounding microenvironmental stimuli and signals (37, 38). It has been recently demonstrated that M2 macrophages can be further subcategorized as M2a, M2b, M2c, or M2d based on the applied stimuli, the resultant transcriptional changes, and their functions (39). Moreover, recent single-cell transcriptome sequencing (scRNA-seq) analyses have shown that synovial monocytes/macrophages of RA patients and RA mouse model are heterogeneous and these distinct subsets are closely linked to diverse homeostatic, regulatory, and inflammatory functions (62–64). Leukocyte-rich RA synovia have a greater abundance of *IL1B* ^+^ monocytes but a reduced *NUPR1* ^+^ monocytes, whereas MerTK^+^ macrophages are associated with remission and maintenance of RA (62, 63). In present study, we found that incubation with activated platelets lead to skewing of monocytes toward differentiation into macrophages with an M2 propensity (**Figure 5**). It is still debated whether platelet interactions with monocytes/macrophages elicit proinflammatory or anti-inflammatory responses upon various stimulations (27, 65–67). Of note, activated platelets alone induce anti-inflammatory responses of monocytes/macrophages *via* PGE_2_ and cytokines (67). In our study, ADP-activated monocytes appear to differentiate into CD16^+^CD163^+^MerTK^+^ M2c macrophages that are polarized by IL-10, TGF-β, or glucocorticoid and play crucial roles in the phagocytosis of apoptotic cells (68). M-CSF is capable of inducing polarization of M2 macrophages that express CD16 and CD163 on monocytes during differentiation (69). However, we found that monocytes pre-treated with ADP-platelets further increased the expression of CD16 and CD163 during differentiation in the presence of M-CSF compared to control. Furthermore, MerTK, a marker of M2c macrophages, was significantly upregulated in HMDMs pretreated with activated platelets. A recent study demonstrated that M2c-like cells are detectable among circulating CD14^+^CD16^+^, but not CD14^dim^CD16^+^, monocytes (70).

In summary, we provide evidence that activated platelets are important for induction of CD16 expression on classical CD14^+^CD16^-^ monocytes through sequential involvement of platelet-derived TGF-β and monocyte-derived IL-6. Induced CD16 on monocytes participates in IgG-mediated phagocytosis. In addition, monocytes pretreated with activated platelets preferentially differentiate into M2c-like macrophages in the presence of M-CSF. In RA patients, the plasma level of sCD62P, a marker of activated platelets, was significantly elevated, which may explain the accumulation of CD14^+^CD16^+^ monocytes in RA patients. Furthermore, the sCD62P level in plasma is positively correlated with RA clinical parameters. These findings underscore a key role of activated platelets for regulating phenotypical and functional features of human monocytes and shed light on the immunological role of CD14^+^CD16^+^ cells in a variety of inflammatory disorders.

## Data Availability Statement

All datasets generated in this study are included in the article/**Supplementary Material**. Further inquiries can be directed to the corresponding author.

## Ethics Statement

The studies involving human participants were reviewed and approved by the Institutional Review Board (IRB) of Seoul National University Hospital and of Chungnam National University Hospital. The patients/participants provided their written informed consent to participate in this study.

## Author Contributions

SL: participated in the design of the study, performed most of the experiments, data collection and analysis, and drafted the manuscript. BY and HK: participated in the design of the study, performed the experiments, data collection and analysis. SK and S-JY: participated in study design and performed data analysis. W-WL: conceived of the study, participated in its design and coordination, performed data analysis and writing of manuscript, and has full access to all the data in this study and financial support. All authors contributed to the article and approved the submitted version.

## Funding

This work was supported by grant (NRF-2018R1A2B2006310 to W-WL) from the National Research Foundation of Korea (NRF) funded by Ministry of Science and ICT (MSIT).

## Conflict of Interest

The authors declare that the research was conducted in the absence of any commercial or financial relationships that could be construed as a potential conflict of interest.
