# Supplementary material for: Activated Platelets Convert CD14+CD16- Into CD14+CD16+ Monocytes With Enhanced FcγR-Mediated Phagocytosis and Skewed M2 Polarization
Source: Front Immunol. 2021 Jan 7;11:611133. doi: 10.3389/fimmu.2020.611133 (PMC7817612; doi:10.3389/fimmu.2020.611133)
Supplement: Supplementary file 1 [file DataSheet_1.docx]

**Activated platelets convert CD14^+^CD16^-^ into CD14^+^CD16^+^ monocytes with enhanced FcγR-mediated phagocytosis and skewed M2 polarization**

Su Jeong Lee, Bo Ruem Yoon, Hee Young Kim, Su-Jin Yoo, Seong Wook Kang, and Won-Woo Lee


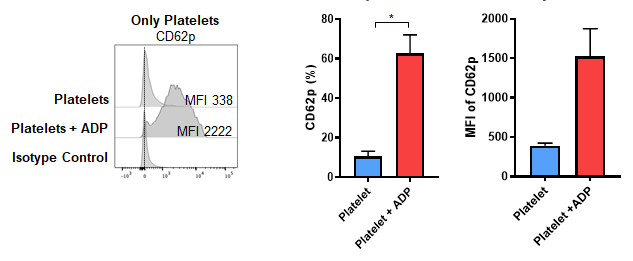


Supplementary Figure 1. Freshly purified platelets are resting state and are activated with ADP. Isolated platelets were treated for 5 min with or without ADP. Platelets were immediately fixed with 4% formalin and stained with anti-CD62P Ab. The expression of CD62P was analyzed using flow cytometry. Bars plots show the mean ± S.E.M. * = *p* < 0.05 by two-tailed paired *t*-test.


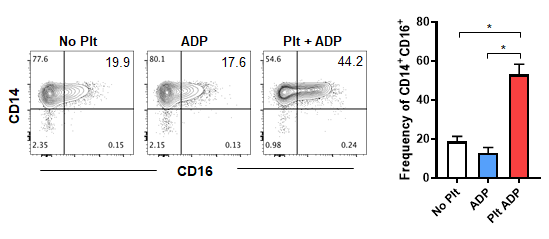


Supplementary Figure 2. No direct effect of ADP on induction of CD16 expression in CD14^+^CD16^-^monocytes. Purified monocyte were stimulated for 18 h with ADP alone or ADP-activated platelets. Induction of CD16 expression was analyzed by flow cytometry. Bars plots show the mean ± S.E.M. * = *p* < 0.05 by two-tailed paired *t*-test.


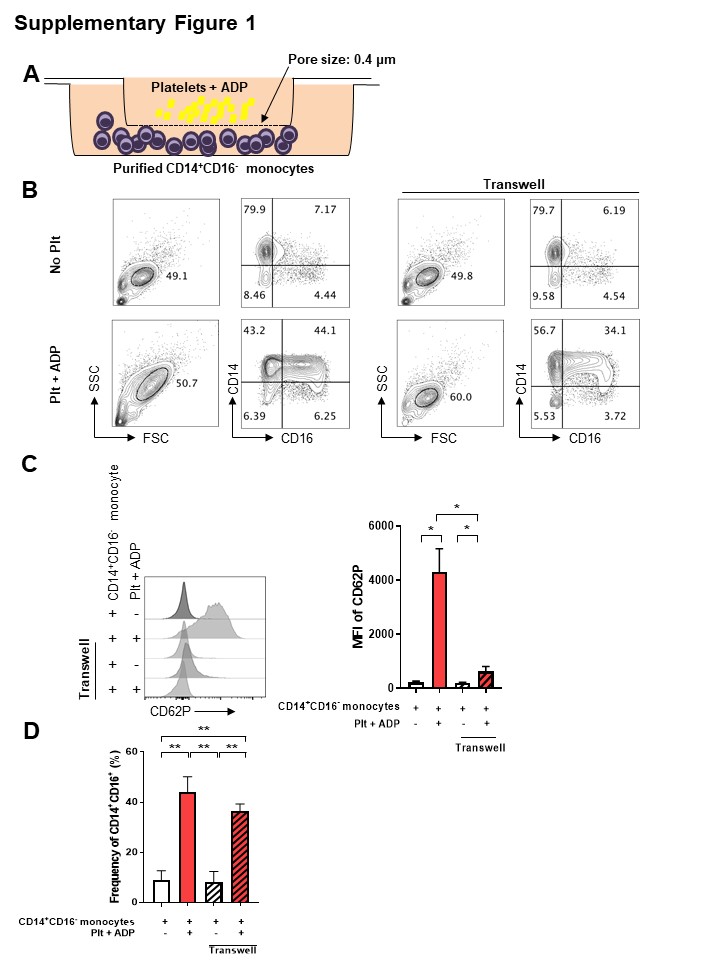


**Supplementary Figure 3.** **Direct contact between monocytes and activated platelets is dispensable for CD16 induction.** For transwell experiments, ADP-activated platelets were added into upper chamber and purified CD14^+^CD16^-^ monocytes were cultured in the lower chamber for 18 h. **(A)** Transwell culture system. **(B)** Representative contour plot of FSC/SSC of monocytes and CD14/CD16 expression on monocytes in the transwell culture system. **(C)** Representative histogram plots of CD62P expression on monocytes under the indicated conditions (left panel). MFI of CD62P in monocytes under the indicated conditions were analyzed using flow cytometry (right panel) (*n* = 3). **(D)** Frequencies (%) of CD14^+^CD16^+^ monocytes under the indicated conditions were analyzed using flow cytometry (lower panel) (*n* = 3). Bars plots show the mean ± S.E.M. * = *p* < 0.05 and ** = *p* < 0.01 by two-tailed paired *t*-test.


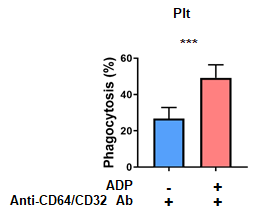


**Supplementary Figure 4.** **Activated platelet-induced CD16 expression on monocytes is involved in CD16-dependent phagocytosis.** Purified CD14^+^CD16^-^ monocytes were stimulated for 18 h with resting platelets or ADP-activated platelets. After that, monocytes were blocked for 30 min by anti-CD64 and anti-CD32 neutralizing antibodies, followed by incubation of 15 min with latex beads coated with FITC-labeled rabbit IgG*.* The phagocytic activity of monocytes was analyzed using flow cytometry. Frequency (%) of monocyte phagocytosis of latex beads coated with FITC-labeled rabbit IgG under the indicated conditions. (*n* = 6). Bars plots show the mean ± S.E.M. *** = *p* < 0.005 by two-tailed paired *t*-test.


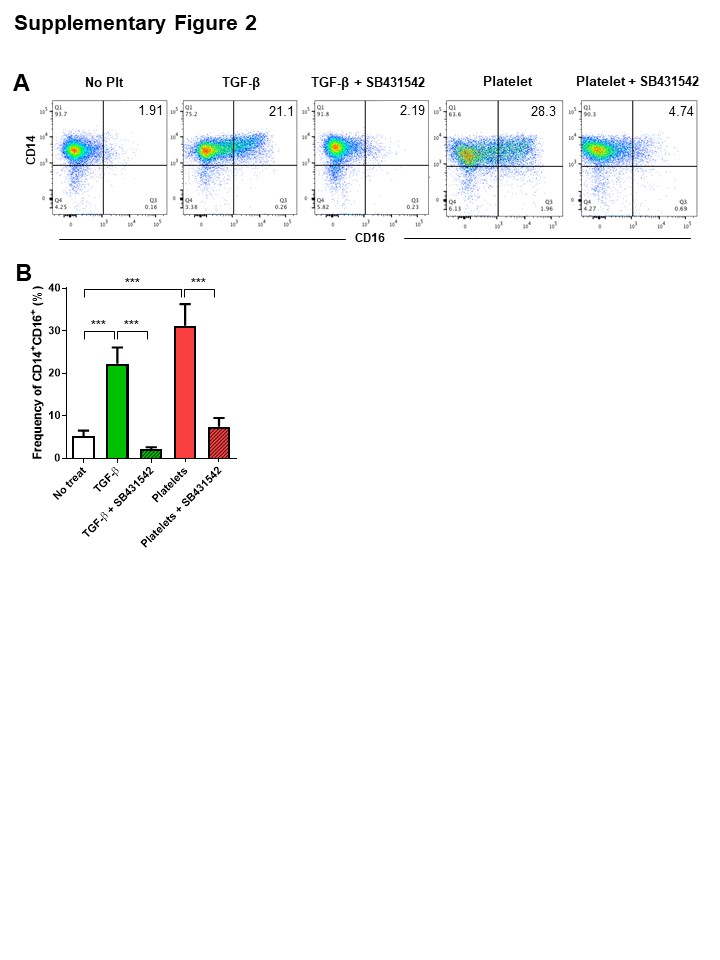


**Supplementary Figure 5.** **Marked inhibition of activated platelet-induced CD16 by the TGF-βRI inhibitor, SB431542.** Purified CD14^+^ CD16^-^ monocytes were pre-treated with SB431542 (1 μg/ml), followed by treatment with TGF-β or ADP-activated platelets for 18 hr. **(A)** Representative contour plots of CD16 expression on monocytes under the indicated conditions. (B) Frequencies (%) of CD14^+^CD16^+^ monocytes under the indicated conditions were analyzed using flow cytometry (*n* = 8). Bars plots show the mean ± S.E.M. *** = *p* < 0.005 by two-tailed paired *t*-test.
